# Influence of different types of whitening tooth pastes on the tooth color, enamel surface roughness and enamel morphology of human teeth

**DOI:** 10.12688/f1000research.20811.1

**Published:** 2019-10-16

**Authors:** Mohamed Shamel, Mahmoud M. Al-Ankily, Mahmoud M. Bakr

**Affiliations:** 1 Oral Biology Department, The British University in Egypt, Cairo, Egypt; 2 School of Dentistry and Oral Health, Griffith University, Mount Gravatt, Queensland, 4222, Australia

**Keywords:** Tooth whitening, Blue covarine, Enamel, tooth color, enamel surface roughness, SEM

## Abstract

**Background:** Tooth whitening usually includes the direct use of gels containing carbamide or hydrogen peroxide on the tooth enamel surface through a wide variety of products formulas. A generally new advancement in whitening of teeth uses the significant importance of the tooth color shift from yellow to blue in delivering a general enhancement in the observation of tooth whiteness. The aim of the current work was to measure the tooth whitening effects, surface roughness and enamel morphology of six different types of blue covarine-containing and blue covarine-free toothpastes using in vitro models.

**Methods: **A total of 70 sound extracted human premolars were randomly and equally divided into seven groups, and each subjected to tooth brushing using different toothpastes. Tooth color and enamel surface roughness were measured before and after the brushing procedure using a white light interferometer, and scanning electron microscopy (SEM) was used to assess tooth surface after the procedure.

**Results:** Toothpaste containing blue covarine resulted in the greatest improvement in tooth color amongst all groups as well as a statistically significant color difference when compared to blue covarine-free toothpaste.  Furthermore, blue covarine-containing toothpaste resulted in fewer morphological changes to the enamel surface. This was confirmed with SEM images that showed smooth enamel surfaces with fine scratches.

**Conclusions: **The results from the present study show that blue covarine containing toothpastes are reliable, effective in tooth whitening and produce less surface abrasion when compared to blue covarine-free toothpastes.

## Introduction

Patients are generally dissatisfied with their present teeth color, as demonstrated by many studies in different regions of the world ^1– 6^. This is most evident from the expanded interest for orthodontic treatment and for using products, either in office or at home, for whitening of teeth ^7^.

Attempts to improve the shade of teeth is presently possible using multiple methods, including professional scaling and prophylaxis performed in the dental clinic, fabrication of tooth crowns and laminate veneers and the use of tooth-whitening toothpastes ^8^. Tooth-whitening formulations work by one of two different methods, either by the elimination of extrinsic tooth stain or by bleaching of the tooth itself ^9^.

Tooth whitening usually includes the direct use of gels containing carbamide or hydrogen peroxide on the tooth enamel surface and are available in a wide variety of products formulas. The peroxide moves into the enamel structure to brighten any internal discoloration and hence making the teeth look whiter. Nonetheless, the efficient delivery of peroxides from the toothpaste to the tooth is multifactorial, including design factors, controlling boundaries and the comparatively limited contact periods during brushing ^9, 10^.

Historically, one of the main components in toothpastes used for whitening has been abrasives, which help to eliminate extrinsic stains as well as prevent their formation. A wide range of other components is usually added to this abrasive system, such as surfactants, calcium chelators, polymers and enzymes; however, evidence indicates that the abrasive is the most crucial component in the toothpastes for stain removal ^11, 12^. Yet, there are worldwide governing limitations on the maximum levels of abrasives allowed in any toothpaste and thus there is some restrictions to how much whitening can be effectively and (most importantly) safely obtained through abrasive advancements ^8^.

To numerically evaluate color, a system was suggested by the International Commission on Illumination (CIE) in 1976 which consisted of three different coordinates designed as L*, a* and b*. Any color is totally identified by three coordinates: L*, a* and b*. L* values range from 0 to 100 and denotes darkness/brightness; a* represents the green–red component, with values ranging from –80 (green) to +80 (red); and b* represents the blue–yellow component, with values ranging from –80 (blue) to +80 (yellow) ^13^.

A generally novel advancement in tooth bleaching utilizes the importance of the tooth color change from yellow to blue (i.e. a decrease of b* values) in obtaining a general enhancement in the observation of whiteness of teeth ^14^. The theory is reinforced by tooth whitening studies that show that the change in color from yellow to blue represents significant evidence of tooth whitening and that a decrease in the value of b* color parameter is a crucial factor for tooth whitening recognition by patients ^15, 16^. Using previous optical theories, a toothpaste containing a blue pigment, named blue covarine, was introduced. This paste applies the blue pigment onto the tooth enamel during daily cleaning procedures using toothbrushes, changing tooth color from yellow to blue. Several clinical and *in vitro* studies confirmed that this increase in bluish color and decrease in yellow color makes the teeth look whiter immediately after tooth brushing ^17, 18^.

Blue covarine-containing tooth pastes have been additionally improved by supplementing the concentration levels of blue pigment, so as to furtherly improve the optical tooth whitening advantage ^14, 19^. The aim of the current study was to measure the tooth whitening effects, surface roughness and enamel morphology of six different types of blue covarine-containing and blue covarine-free toothpastes using *in vitro* models.

## Methods

### Samples

A total of 70 sound extracted human premolar teeth, obtained for purposes of research with informed consent. The study was approved by the Research Ethics Committee at Suez Canal University (approval number Suez- REC 27/2018), where this research was performed. The enamel surfaces were then cleaned with prophylactic paste to ensure elimination of extrinsic stains. The teeth were kept in sterilized artificial saliva for two hours. Teeth were mounted in gypsum blocks by embedding the roots and the lingual half, where the middle part of the middle one-third of the buccal aspect is the highest area of the specimen.

The specimens (n= 10 per group) were randomly distributed to the tested toothpastes which were as follows:

Group I: Close up White now (Unilever, São Paulo, Brazil) (with blue covarine)
Group II: Sensodyne True White (GSK, UK) (whitens by abrasion/sodium triphosphate)
Group III: Colgate Optic White (Colgate-Palmolive, USA) (whitens by abrasion/sodium monophosphate with hydrogen peroxide)
Group IV: Close Up (Unilever, São Paulo, Brazil)
Group V: Sensodyne (GSK, UK)
Group VI: Colgate (Colgate-Palmolive, USA)
Group VII: Control: No tooth paste application

Groups I and IV have the same formulation (sodium fluoride), but the former has an additional whitening ingredient. The same is true for groups II and V (potassium nitrate), and III and VI have the same formulation (sodium fluoride), but the former have an additional whitening component. Thus, groups IV, V and VI serve as non-whitening controls for groups I, II and III, respectively.

### Color measurement

The three parameters of color of each specimen were measured using a VITA Easyshade spectrophotometer Advance 4.01 (VITA Zahnfabric, Bad Sackingen, Germany) according to the CIE L*a*b* color order system. Mean measurements of the middle part of the middle one-third of the buccal aspect were recorded.

### Surface roughness measurement

The Baseline surface roughness was studied using Zygo Maxim GP-200 white light interferometer (Artisan Technology Group, Illinois, USA) with a surface area 2 mm x 2 mm. Surface roughness was quantified as deviation from the ideal surface in µm using white light interferometer. Mean measurements of the middle part of the middle one-third of the buccal aspect were recorded.

### Brushing procedures

**Figure 1.**
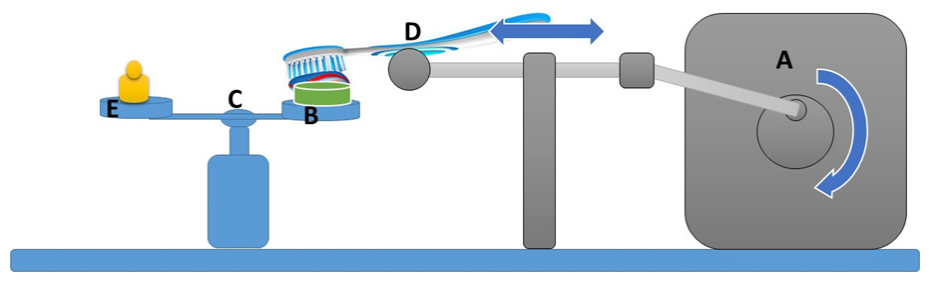
Diagrammatic drawing showing the brushing apparatus. The Brushing apparatus consists of: ( **A**) a gear box to reduce the speed of the motor to 2 cycle/second with a crankshaft and connecting rod attached to a slider in order to change the rotation movement into linear movement to provide a standardized 5 mm horizontal movement; ( **B**) a brush holder; ( **C**) double-pane balance; ( **D**) samples holding pane; and ( **E**) weight holding pane.

For the purpose of standardization of the brushing procedure, a specially designed brushing apparatus was designed and fabricated ( Figure 1). The brushing apparatus was set at 120 strokes/min, and a load of 250 g. For each experimental group, the specimens were fixed in the machine and brushed with a toothpaste corresponding to its group for 3.5 minutes, simulating 4 weeks of brushing three times a day. Every toothpaste was mixed with distilled water with a ratio of toothpaste: water 2:1 ^15^. After tooth brushing, every specimen was rinsed in water to ensure removing all the toothpaste, and the teeth color was remeasured before they were restored in artificial saliva.

### Color measurement difference

The change in color (ΔE*) was calculated as follows:

ΔE*=[(ΔL*) ^2^+(Δa*) ^2^+(Δb*) ^2^]1/2

where ΔL*, Δa*, and Δb* are the difference between the final and initial L*, a*, and b* color parameters, respectively.

### Surface roughness difference

Surface roughness measures after the brushing procedure were recorded and the difference between the baseline was calculated.

### Scanning electron microscopy (SEM) analysis

Surface Morphology was studied using images obtained from SEM of one sample from each group after the brushing procedure. The specimens were rinsed ultrasonically with water for ten minutes and prepared for SEM (FEI, QUANTA FEG 250) analysis. After dehydration, enamel surfaces were sputter-coated with gold (approximately 30–35 nm) and photomicrographs of representative areas were taken at 1000x and 2000x magnifications. The classification for the enamel changes were as follows; no alterations, mild or slight alterations, significant alterations and loss of superficial structures.

### Statistical analysis

All data was analyzed with the statistical program SPSS version 21 (SPSS Armonk, NY, USA). Data from baseline as well as from final measurements were analyzed using one-way analysis of variance (ANOVA) when there is only one factor, which is the type of toothpaste, and Tukey’s test as a post hoc test. A paired Student’s t-test was used to compare the initial and final color parameters for each experimental group.

## Results

### Teeth color

**Figure 2.**
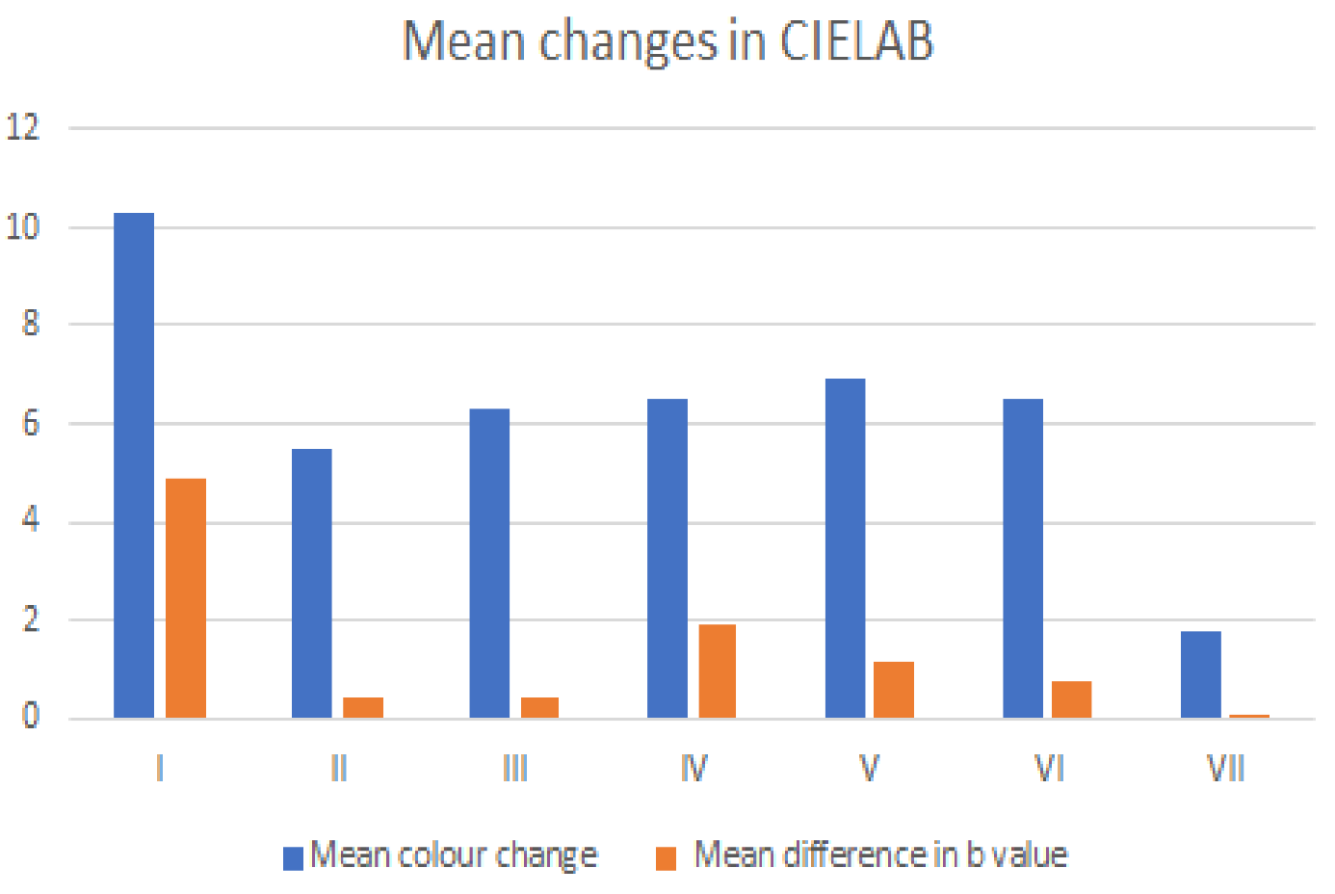
Showing mean changes in color values directly after toothbrushing as well as difference in b* value.

Results for initial and final color parameters for all specimens of all groups are tabulated in Table 1. One-way ANOVA revealed mean color changes which was statistically significant between groups (p=0.01). The mean changes in color parameters which were informed by the International Commission on Illumination (CIELAB) values directly after brushing as well as difference in b value alone showed that toothpaste containing blue covarine (Group I, Close Up White Now) gave the highest decrease in b*, revealing a bluish change in the color of the teeth and thus the highest improvement in tooth whiteness correspondingly, while the control group showed the least reduction in b* and tooth whiteness ( Figure 2).

**Table 1.** Color parameters for specimens of all groups before and after brushing.

Toothpaste	Sample	Before			After		
		L*	a*	b*	L*	a*	b*
**Closeup** **white now**	A1	83.7	-1.9	20.8	84.8	-2.2	18.6
	A2	75.8	-1.5	19.6	77.3	-2.3	16.5
	A3	79.8	1.4	26.1	73.9	0.5	22.6
	A4	83.6	0.9	40.5	86.1	0.2	33.2
	A5	83.1	-2.3	30.5	80.1	-1.2	30.4
	A6	87.3	-1.4	21.8	94.6	-2.8	21.5
	A7	73.7	0.3	27.8	77.4	-2.2	18.8
	A8	79.8	1.3	27.3	77.2	1	25.4
	A9	82.1	0.9	40.1	76.3	0	21.3
	A10	84.6	-1.4	33.5	81.2	-1.7	30
**Sensodyne** **true white**	B1	71.5	-2.6	16	72.8	-1.4	23.1
	B2	86.4	-1.7	22.7	79.4	-1.6	20.1
	B3	81.3	3.5	42.8	79.8	1.5	33.2
	B4	76	1	27.3	72.2	4.1	35
	B5	86.3	0.4	31	83	0.5	30.6
	B6	77.8	-1.8	20.9	71.9	-1.4	22.5
	B7	79	-1.1	17.4	79.3	-0.9	17.5
	B8	80.6	-5	27.3	77.9	-0.1	23.1
	B9	82.4	-0.9	23.4	82.1	-1.2	22.1
	B10	82.5	-0.2	26.3	85.2	-0.3	28.1
**Colgate** **optic white**	C1	78.9	-1.1	23.2	81.7	-1.2	25.1
	C2	76.9	-0.4	24.7	76.3	-0.4	25.8
	C3	82.2	-0.6	31.8	78.9	-0.5	26.2
	C4	78.7	-0.3	30.2	71.7	-2.2	14.9
	C5	80.7	-1.1	18.3	80.2	-1.7	15
	C6	87.4	-0.9	27.3	86.2	-1	27.2
	C7	79.6	-0.8	24.8	82.8	-0.9	26.3
	C8	78.3	-2.2	17.8	81.9	-1.6	27.9
	C9	82.3	-1.7	19.6	69.7	-1.2	22.2
	C10	78.7	-1.1	18	79.1	-0.7	20.7
**Sensodyne**	E1	89.6	-1.9	26.1	79.8	-1.9	25.7
	E2	80.5	-0.6	25.9	75.9	-0.8	23.2
	E3	79.1	-2.9	5.5	85.1	-3.1	8.8
	E4	69.2	-0.3	27.2	66.6	-0.2	27.3
	E5	86.2	1.6	32.9	73.6	1.9	31.1
	E6	85.8	-2.6	19	78.3	-2.3	16.8
	E7	78	0.3	25.1	70	-0.4	20.4
	E8	80.2	-2.3	12	81.6	-1.9	14.3
	E9	75.7	-1.5	20	70.9	-1.7	16.8
	E10	78.5	-0.3	20.8	71.9	0	18.7
**Colgate**	F1	86.3	-2.2	24.3	89.9	-1.5	33.2
	F2	83.2	-0.2	35.9	82.9	0.1	34.7
	F3	84.6	-2.2	14.6	81.8	-1.8	18
	F4	86.4	-2.9	13	91.7	-3.2	18.3
	F5	87.8	3	35.4	78.5	2.9	32.3
	F6	87.9	-1.3	35.5	89.4	-1.3	33.2
	F7	84.8	0	39.7	84.8	-0.1	36.9
	F8	80.3	-0.2	29.7	79.9	-2.1	15.8
	F9	89.1	-2.7	22.4	87.5	-1.6	27.4
	F10	77.6	4.3	37	77.2	2	30.1
**Control**	G1	74.6	2.7	35.2	76.2	2.4	35.6
	G2	66.9	1.9	29	67.7	4.7	40.5
	G3	69.9	2.9	34.7	63.4	3.2	32
	G4	87.1	-1.5	19.5	79.1	-1.8	20.7
	G5	82.7	-2	14.2	82.1	-1.4	22.9
	G6	81.6	-1.3	21.2	84.8	-1.8	17.6
	G7	81.4	-0.3	27.2	80.6	-0.1	29.7
	G8	83.9	-0.7	26.5	78.1	-2.5	18.2
	G9	82.2	-1.2	23.6	81.9	-2	22.3
	G10	79.1	1.1	21.6	77.5	-1.2	20.4

Toothpaste which contained blue covarine (Group I) had a color statistically significant difference compared with the non-whitening control, group IV (Close up regular tooth paste) (p = 0.02, Student’s t-test) and also a statistically significant difference with other types of whitening tooth pastes not containing blue covarine group II (p=0.005) and group III (p=0.04), . Mean color changes of specimens treated with whitening blue covarine-free tooth pastes (groups II and III) showed a positive but statistically non-significant increase in the overall color change and a slight increase in the b* when compared to their non-whitening controls, groups V and VI, respectively (p = 0.08, Student’s t-test). Raw tooth color values for each group are available as *Underlying data* ^20^.

### Surface roughness

**Figure 3.**
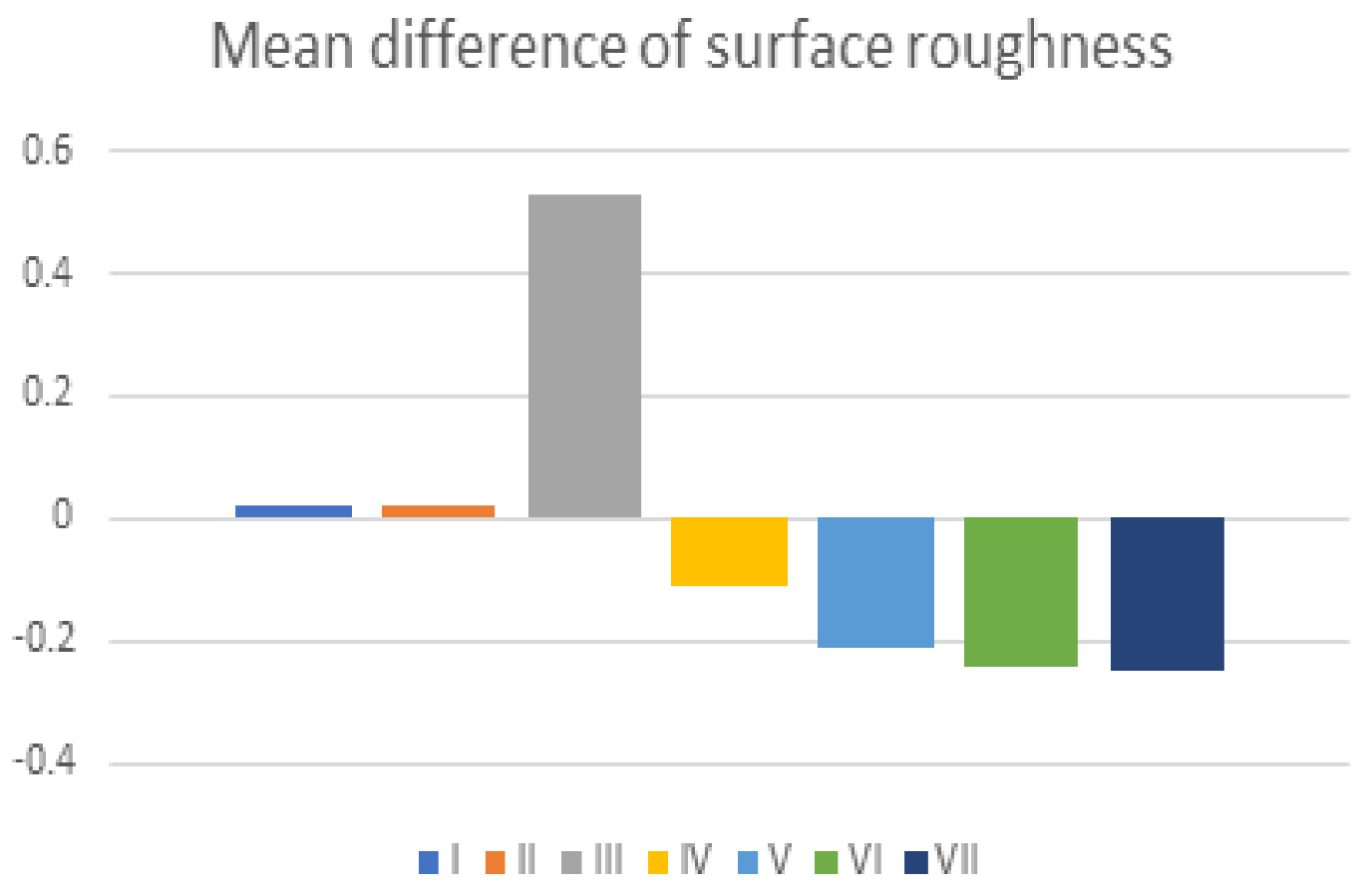
Mean differences in surface roughness of all groups.

**Figure 4.**
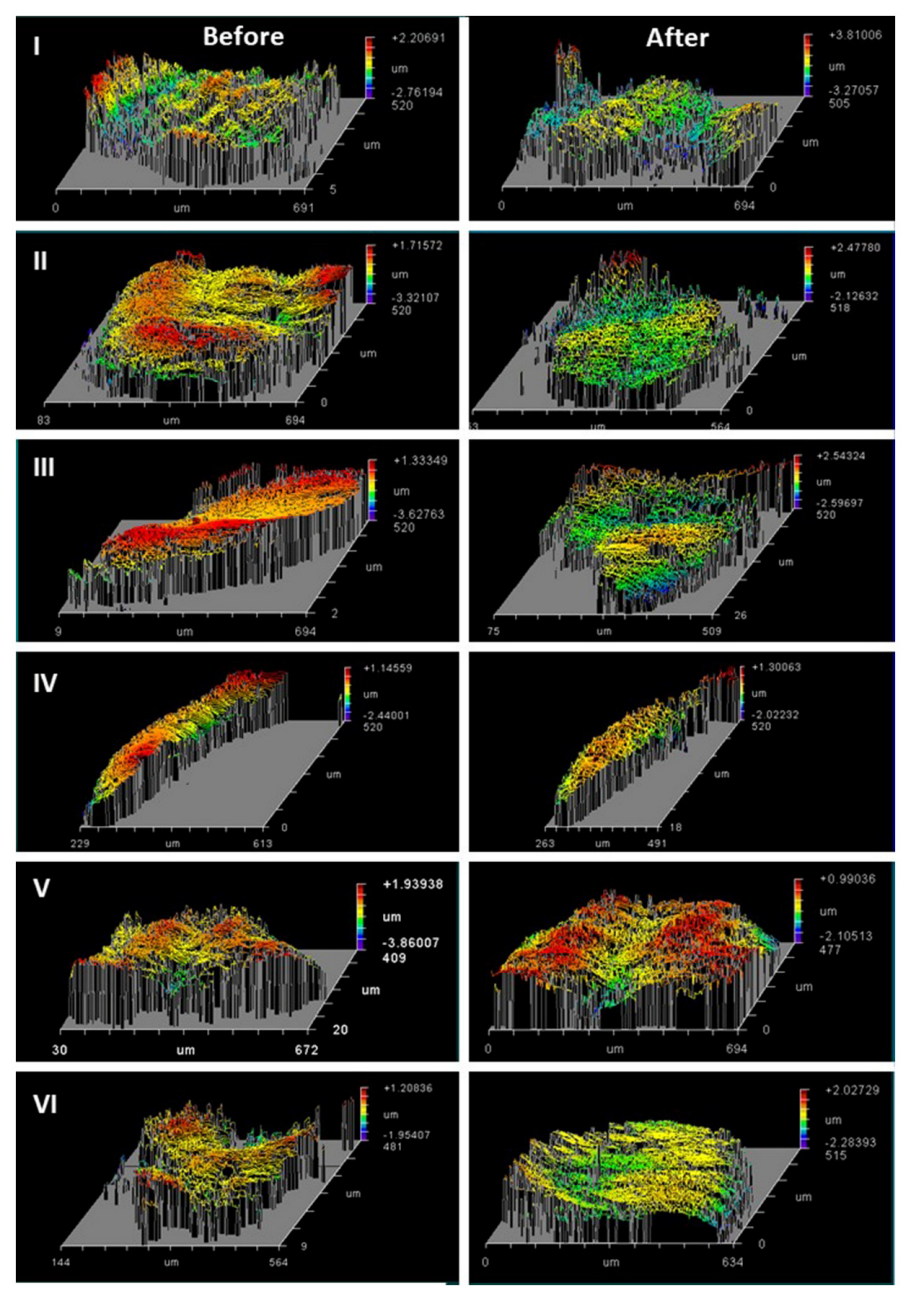
White light interferometer images of all groups before and after brushing procedure.

Results of surface roughness measurement of specimens of all groups are tabulated in Table 2. There were no statistically significant differences in the mean values of surface roughness difference (Ra) of all groups (p= 0.07). The control group showed the least surface roughness change, with a reduction in median Ra of 0.25 μm; the treatment with the toothpaste containing blue covarine (Group I) showed Ra value of 0.02 μm; the Ra value of tooth paste of group II was 0.02 μm while that of group III showed the highest Ra value of 0.53 ( Figure 3 and Figure 4). Raw surface roughness values and white light interferometry images are available as *Underlying data* ^20, 21^.

**Table 2.** Surface roughness measurements before and after brushing for specimens of all groups.

Toothpaste	Sample	Average roughness (µm) BEFORE	Average roughness (µm) AFTER	Difference
**Closeup** **white now**	A1	1.34	1.15	-0.19
	A2	0.08	0.18	0.10
	A3	0.48	0.36	-0.12
	A4	0.11	0.13	0.02
	A5	1.10	1.22	0.12
	A6	0.19	0.18	-0.01
	A7	0.67	0.62	-0.05
	A8	0.15	0.35	0.20
	A9	1.01	1.06	0.05
	A10	0.07	0.19	0.12
**Sensodyne** **true white**	B1	0.08	0.29	0.21
	B2	0.38	0.32	-0.06
	B3	0.42	0.39	-0.03
	B4	0.17	0.19	0.02
	B5	0.07	0.30	0.23
	B6	0.37	0.32	-0.05
	B7	0.49	0.42	-0.07
	B8	0.22	0.21	-0.01
	B9	0.27	0.28	0.01
	B10	0.37	0.31	-0.06
**Colgate** **optic white**	C1	0.64	0.74	0.10
	C2	0.14	0.49	0.35
	C3	0.14	0.09	-0.05
	C4	0.27	0.23	-0.04
	C5	0.67	0.88	0.21
	C6	0.13	0.49	0.36
	C7	0.08	0.70	0.62
	C8	0.29	0.31	0.02
	C9	0.74	0.88	0.14
	C10	0.12	0.53	0.41
**Closeup**	D1	0.38	0.45	0.07
	D2	0.37	0.22	-0.15
	D3	1.27	0.49	-0.78
	D4	0.74	0.54	-0.20
	D5	0.38	0.45	0.07
	D6	0.30	0.18	-0.12
	D7	1.11	1.22	0.11
	D8	0.75	0.73	-0.02
	D9	0.47	0.54	0.07
	D10	0.30	0.18	-0.12
**Sensodyne**	E1	0.31	0.35	0.04
	E2	1.87	0.73	-1.14
	E3	0.43	0.42	-0.01
	E4	0.30	0.25	-0.05
	E5	0.35	0.34	-0.01
	E6	2.04	0.60	-1.44
	E7	0.18	0.29	0.11
	E8	0.30	0.25	-0.05
	E9	0.26	0.33	0.07
	E10	1.23	0.72	-0.51
**Colgate**	F1	1.10	0.87	-0.23
	F2	0.96	0.98	0.02
	F3	0.25	0.24	-0.01
	F4	0.47	0.48	0.01
	F5	1.30	0.89	-0.41
	F6	0.78	1.06	0.28
	F7	0.26	0.19	-0.07
	F8	2.28	0.36	-1.92
	F9	1.30	0.91	-0.39
	F10	0.76	1.05	0.29
**Control**	G1	1.00	1.43	0.43
	G2	0.83	0.26	-0.57
	G3	0.88	0.44	-0.44
	G4	0.24	0.35	0.11
	G5	1.17	1.63	0.46
	G6	0.97	0.23	-0.74
	G7	0.85	0.46	-0.39
	G8	0.21	0.35	0.14
	G9	1.60	1.55	-0.05
	G10	1.85	0.40	-1.45

## SEM examination

**Figure 5.**
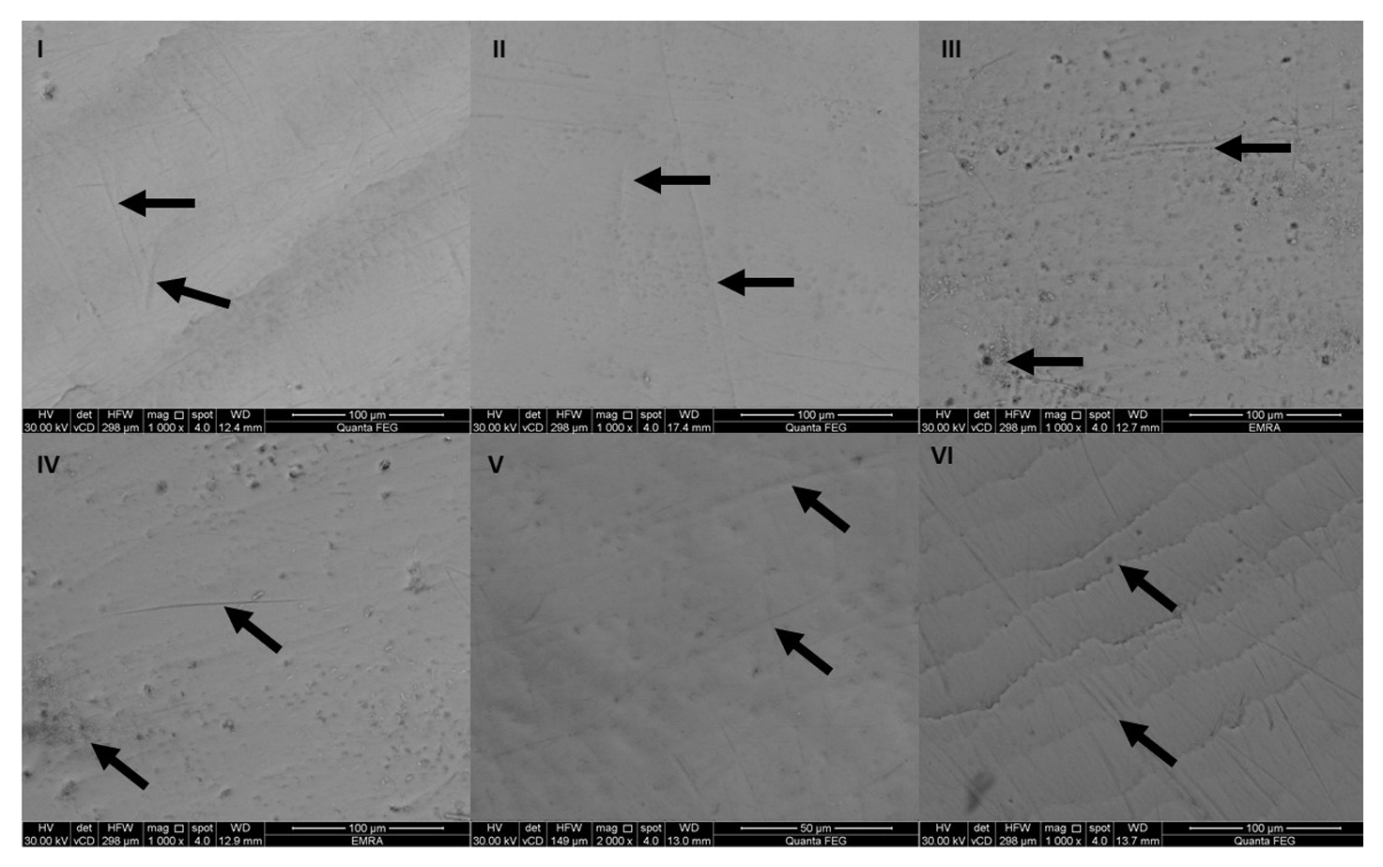
SEM micrographs of experimental groups after brushing procedure. Groups I and II show smooth enamel surface with fine scratches (arrows). Group III shows irregular enamel with deeper surface scratches and pores (arrows). Groups IV, V and VI show fine surface scratches and few pores (arrows).

Group I tooth samples revealed relatively smooth enamel surface with few fine scratches. Group II showed similar surface pattern with some fine scratches on the enamel surface when compared to control group, whereas tooth paste of group III caused greater alteration in enamel morphology manifested as surface irregularities, few pores and also the scratches became more obvious. Samples from groups IV, V and VI exhibited no noticeable differences regarding the morphology of the surface of the enamel in comparison to the control samples ( Figure 5). Raw SEM images are available as *Underlying data* ^21^.

## Discussion

A variety of commercially available toothpastes with and without bleaching agents for home use were chosen for this study to evaluate color change, surface roughness and surface structure of tested teeth. The findings of this study revealed toothpastes containing blue covarine preparations had a measurable improvement in tooth whiteness which was significantly increased directly after toothbrushing in comparison to other products not containing blue covarine, including group II and III toothpastes, which showed a statistically significant color difference, (p=0.005 and p=0.04, respectively).

 In the present work, it was revealed that the blue covarine-containing toothpaste was the only toothpaste tested to give a significant decrease in b* value and improvement in tooth whiteness (∆E) in comparison to the controls. This decrease in b* value is mainly due to the deposition of the pigments on the enamel surface, which causes modification to the optical characters of the tooth surface ^22^. The use of the blue pigment in toothpastes changes the observation of the yellow color in teeth by applying a thin, translucent blue layer on the tooth surface, thus creating a optical whitening effect. Yellow is opposite to blue in the spectrum of colors, thus a change or shift to blue creates an appearance which visually appears whiter by changing the net color towards white. This mechanism of whitening is built on many previous studies performed which showed that the blue shift of the yellow–blue axis (b*) is of higher significance in the observation of whiter teeth than other axis, (L*) or (a*) ^23– 25^.

There are contradictory results in the literature regarding the efficiency of blue covarine to provide an improved whitening result in teeth whiteness. Many past and recent studies showed instant and progressive whitening ^14, 19, 26– 28^; however, some other studies reported no whitening effect of the blue covarine formulations ^29, 30^. This contradiction in results might be due to using different techniques in recording the color change following brushing procedure which includes the use of either visual or instrumental tools. In the present study, a Vita Easyshade spectrophotometer was used to measure the color change as it proved in several studies to be more accurate than visual tools ^31, 32^.

In contrast, all other tooth pastes tested in this study had a relatively no significant change in b* and overall color change values (∆E) when compared to the control groups. In this study, the tested toothpaste with 1% hydrogen peroxide (Colgate White now) presented a whitening effect similar to that of conventional toothpaste because it contains abrasive particles in the composition. During toothbrushing, the abrasive particles are trapped between the tips of the toothbrush bristles and the stained tooth surface. This was confirmed by results from the surface roughness experiment, where group III specimens had the highest increase in mean difference of surface roughness. Since the abrasive particles are physically harder than the superficial staining, this is removed leaving a clean tooth surface. Thus, the abrasive cleaning mechanism mainly influences the extrinsic stains and does not significantly affect any underlying intrinsic staining or the natural color of teeth ^33, 34^. This finding is consistent with previous studies that concluded that conventional dentifrices could outperform or have a similar whitening effect to whitening toothpaste ^30, 35, 36^.

 Concerning enamel surface roughness, Atomic force was not used to measure the surface roughness due to its limited measurement of an area of 25 μm on a flat area only, whereas a white light interferometer was used in this study due to its wider area measurement (2 mm x 2 mm) and ability to assess non-flat surfaces, which is superior to the properties of atomic force.

 Although there were no statistically significant differences between all groups in our study, group III toothpaste created the roughest surface, while group VI toothpaste resulted in the smoothest surface in comparison to control group. This change in physical properties can occur due to effects of demineralization and remineralization of enamel surface ^37^. This demineralization can be caused by diffusion of hydrogen peroxide found in the toothpaste in group III.

 The previous results of color change and surface roughness are consistent with the study performed by Vieira-Junior *et al*., which showed that the alteration in surface roughness has no significant correlation with b* values or significant alteration in the general color change represented by ∆E values ^38^.

 In the present study, it was aimed to compare the changes caused by different toothpastes with different compositions on human enamel surface with SEM and visualize the structural changes with photomicrographs. Specimens of the control group revealed a normal enamel surface, while that of groups I and II showed minor changes of the enamel surfaces manifested as fine scratches. However, group III specimens showed some alteration in the enamel surface manifested as surface irregularities, few pores and the scratches became more obvious.

## Conclusion

From the results of the current study it can be considered that toothpastes with blue covarine are both an effective and a safe method to improve the whiteness of teeth in the routine home tooth brushing.

## Data availability

Figshare: tooth color (Influence of different types of whitening tooth pastes on the tooth color, enamel surface roughness and enamel morphology of human teeth). https://doi.org/10.6084/m9.figshare.9923756.v1 ^20^.

This project contains the following underlying data:

- tooth color (Influence of different types of whitening tooth pastes on the tooth color, enamel surface roughness and enamel morphology of human teeth).xlsx
- surface roughness (Influence of different types of whitening tooth pastes on the tooth color, enamel surface roughness and enamel morphology of human teeth).xlsx

Figshare: Influence of different types of whitening tooth pastes on the tooth color, enamel surface roughness and enamel morphology of human teeth. https://doi.org/10.6084/m9.figshare.9923780.v1 ^21^.

This project contains raw scanning electron microscopy and white light interferometry images for each group.

Data are available under the terms of the Creative Commons Attribution 4.0 International license (CC-BY 4.0).
