# Supplementary material for: Influence of different types of whitening tooth pastes on the tooth color, enamel surface roughness and enamel morphology of human teeth
Source: F1000Res. 2019 Oct 16;8:1764. [Version 1] doi: 10.12688/f1000research.20811.1 (PMC6900807; doi:10.12688/f1000research.20811.1)
Supplement: Peer review history

# Peer review: Reviewer response for version 1

**DOI:** 10.5256/f1000research.22891.r55277

**Published:** 2019-12-06

**Authors:** Rizwan Ullah

**Affiliations:** 1 Sindh Institute of Oral Health Sciences, Jinnah Sindh Medical University, Karachi, Pakistan

- The title of the study is quite lengthy. It is suggested to the authors to reduce the length of article title.
- Does the blue covarine toothpaste have any additional whitening agent in its composition?
- In methods, instead of sterilized artificial saliva write "sterile saliva".
- In Figure 1, please recheck the labelling, especially B and D.
- In the discussion, give a brief account of the limitations of your study.

Overall the study was well performed and showed some interesting results. The standardized brushing method was a very good way to ensure the accuracy of the results of the experiment. Statistical analysis of data is available and appropriately interpreted. However some minor suggestions could be taken into consideration: Further analysis between the results of different groups, e.g group II, III and IV could have added to the study. Overall, the study was clear and well written.

I confirm that I have read this submission and believe that I have an appropriate level of expertise to confirm that it is of an acceptable scientific standard.

# Peer review: Reviewer response for version 1

**DOI:** 10.5256/f1000research.22891.r57522

**Published:** 2019-12-06

**Authors:** Mahmoud Serag

**Affiliations:** 1 Prosthodontics Division, General Dentistry Department, School of Dental Medicine, East Carolina University (ECU), Greenville, NC, USA

The present study investigated the effect of different types of tooth pastes on the enamel surface properties.

 The introduction section was informative, brief and well written. The authors highlighted the numerical evaluation of color as well as the different methods and materials for tooth whitening using tooth pastes at home.

 The white light inteferometer used to measure surface roughness is an accurate method which ensured reliable results. The same applies to the brushing apparatus used although it was not clear the type of tooth brush used nor the frequency of changing the tooth brush.

 Different formulations of the investigated tooth pastes were not fully written.

 The results were clear and the SEM micrographs were explained thoroughly. However, more statistical analysis could have been added to compare between more experimental groups, not only with the control.

 The discussion was written well and explained the outcomes of the research.

 Overall the present research was written and executed well with clear results.

I confirm that I have read this submission and believe that I have an appropriate level of expertise to confirm that it is of an acceptable scientific standard.

# Peer review: Reviewer response for version 1

**DOI:** 10.5256/f1000research.22891.r55473

**Published:** 2019-10-22

**Authors:** Rania Mossad Hassan

**Affiliations:** 1 Faculty of Dentistry, Ain Shams University, Cairo, Egypt; 2 Badr University, Cairo, Egypt

This study investigated the effect of different whitening toothpastes on the teeth enamel color surface roughness and morphology.

 The topic is interesting and the manuscript is well written and well constructed. The introduction was informative and covered the main ideas investigated in the study.

 The apparatus used to simulate the brushing provided a standardized procedure thus aiding in having accurate results.

 All data of teeth color and surface roughness are available.The discussion was well written and covered and explained the results thoroughly.

 However, some minor comments are suggested for the authors to consider:

- More than one type of tooth pastes with blue covarine could have been tested to compare their efficacy, or different concentrations of blue covarine.
- The authors didn`t mention if a new toothbrush was used in the experiment for each tooth or not.
- In figure 2 the word "colour" is different than the spelling in the rest of the research.

I confirm that I have read this submission and believe that I have an appropriate level of expertise to confirm that it is of an acceptable scientific standard.
